# Bis(2-{[2-(2-hy­droxy­benzyl­amino)­eth­yl]amino­meth­yl}phenolato-κ^3^ *N*,*N*′,*O* ^1^)cobalt(III) nitrate monohydrate

**DOI:** 10.1107/S1600536811055851

**Published:** 2012-01-07

**Authors:** Mouhamadou Moustapha Sow, Ousmane Diouf, Ibrahima Elhadj Thiam, Mohamed Gaye, Pascal Retailleau

**Affiliations:** a Département de Chimie, Faculté des Sciences et Techniques, Université Cheikh Anta Diop, Dakar, Senegal; b ICSN-CNRS, Laboratoire de Cristallochimie, 1 Avenue la Terasse, 91198 Gif-sur-Yvette, France

## Abstract

In the title compound, [Co(C_16_H_19_N_2_O_2_)_2_]NO_3_·H_2_O, the Co^III^ ion is located on an inversion center and is six-coordinated by two phenolate O atoms and four amino N atoms from two diamine ligands, forming an octa­hedral geometry. The water mol­ecule and the nitrate anion are located close to an inversion center, and are thus equally disordered by symmetry. The crystal packing is stabilized by inter­molecular O—H⋯O hydrogen bonds involving the uncoordinated water mol­ecule and the free phenol hydroxyl group with the nitrate anion. N—H⋯O hydrogen bonds involving the amino groups and the nitrate anions connect the complex mol­ecules along the *c* axis.

## Related literature

For related structures, see: Zhou (2009 ▶); Zhang (2010 ▶); Khalaji *et al.* (2010 ▶).
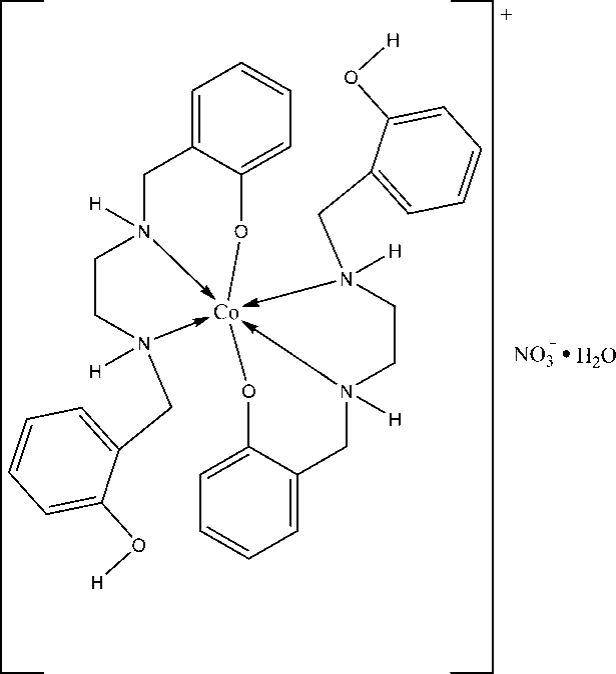


## Experimental

### 

#### Crystal data

[Co(C_16_H_19_N_2_O_2_)_2_]NO_3_·H_2_O
*M* *_r_* = 681.62
Triclinic, 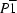

*a* = 8.989 (3) Å
*b* = 9.032 (3) Å
*c* = 10.621 (4) Å
α = 106.680 (2)°
β = 99.950 (3)°
γ = 109.720 (2)°
*V* = 742.0 (4) Å^3^
*Z* = 1
Mo *K*α radiation
μ = 0.64 mm^−1^
*T* = 293 K
0.42 × 0.14 × 0.12 mm

#### Data collection

Nonius KappaCCD diffractometer
Absorption correction: multi-scan (*SCALEPACK*; Otwinowski & Minor, 1997 ▶) *T*_min_ = 0.640, *T*_max_ = 0.930
16124 measured reflections
2694 independent reflections
2303 reflections with *I* > 2σigma(*I*)
*R*_int_ = 0.019

#### Refinement

*R*[*F* ^2^ > 2σ(*F* ^2^)] = 0.047
*wR*(*F* ^2^) = 0.126
*S* = 1.10
2689 reflections
234 parameters
29 restraints
H-atom parameters constrained
Δρ_max_ = 0.25 e Å^−3^
Δρ_min_ = −0.48 e Å^−3^

### 

Data collection: *COLLECT* (Nonius, 1998 ▶) and *DENZO* (Otwinowski & Minor, 1997 ▶); cell refinement: *COLLECT* and *DENZO*; data reduction: *SCALEPACK* (Otwinowski & Minor, 1997 ▶); program(s) used to solve structure: *SHELXS97* (Sheldrick, 2008 ▶); program(s) used to refine structure: *SHELXL97* (Sheldrick, 2008 ▶) and *CRYSTALBUILDER* (Welter, 2006 ▶); molecular graphics: *PLATON* (Spek, 2009 ▶) and *Mercury* (Macrae *et al.*, 2006 ▶); software used to prepare material for publication: *SHELXL97*.

## Supplementary Material

Crystal structure: contains datablock(s) I, global. DOI: 10.1107/S1600536811055851/bh2406sup1.cif

Structure factors: contains datablock(s) I. DOI: 10.1107/S1600536811055851/bh2406Isup2.hkl

Additional supplementary materials:  crystallographic information; 3D view; checkCIF report

## Figures and Tables

**Table 1:** Selected geometric parameters (Å, °)

Co1—O1	1.896 (2)
Co1—N1	1.950 (2)
Co1—N2	1.997 (2)

Symmetry code: (i) 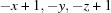.

**Table 2:** Hydrogen-bond geometry (Å, °)

*D*—H⋯*A*	*D*—H	H⋯*A*	*D*⋯*A*	*D*—H⋯*A*
O21—H21*O*⋯O12^ii^	0.95	2.27	3.025 (12)	136
O21—H22*O*⋯O13^ii^	0.95	2.18	2.922 (13)	134
O2—H2*O*⋯O21^iii^	0.82	1.99	2.787 (9)	165
O2—H2*O*⋯O11^iii^	0.82	2.01	2.823 (9)	169
N1—H1*N*⋯O12^iv^	0.86	2.35	3.185 (10)	165
N1—H1*N*⋯O13^i^	0.86	2.31	3.145 (11)	163

Symmetry codes: (i) 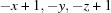; (ii) 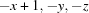; (iii) 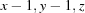; (iv) 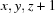.

**Fig. 1.**
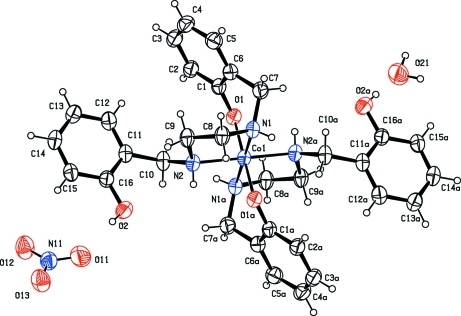
An ORTEP view of the title compound, showing displacement ellipsoids at the 50% probability level. [Symmetry operator a: -x + 1, -y, -z + 1]. A single position for nitrate and water groups has been retained.

**Fig. 2.**
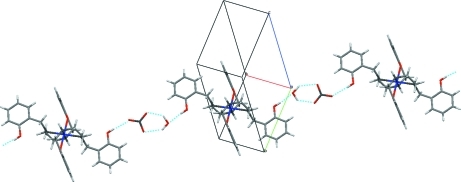
Molecular representation of the compound showing hydrogen bonds. Broken lines stand for hydrogen bonds.

## supplementary crystallographic information

### Comment

Schiff bases with N_2_O_2_ inners have been used to synthesize complexes, due to their chelating ability for metal atoms. In this paper, the title new cobalt(III) complex with the diamine ligand [2-((2-hydroxybenzylamino)ethylamino)methyl]phenol, is reported. The Co^III^ ion is six-coordinated by four amino N atoms and two phenolate O atoms from two ligands, forming an octahedral geometry (Fig. 1). The ligands present one of the hydroxyl groups protonated while the coordinated one is deprotonated. The equatorial sites are occupied by two amino N atoms and two phenolate O atoms from the same arm of the ligand and the two axial sites are occupied by the amino N atoms owned by the arm bearing the non deprotonated phenol group. The Co—O and Co—N equatorial bond lengths are 1.896 (2) and 1.950 (2) Å, while Co—N axial bond lengths is 1.997 (2) Å (Table 1). They are comparable to the bond lengths in similar octahedral cobalt complexes (Zhou, 2009; Zhang, 2010; Khalaji *et al.*, 2010). The NO_3_^-^ ion and the lattice water molecule are disordered over two sets of sites, with relative occupancies of 0.5 for each group. In the crystal structure, the molecules of the compound are linked into a three-dimensional framework by a combination of O—H···O, N—H···O, and C—H···π(arene) hydrogen bonds and also π–π stacking interactions. The first hydrogen bond type utilizes hydroxyl atoms O2 as donors to link the Co complexes through either a water molecule or a nitrate ion (the water molecule being H-bonded to the nitrate ion over a crystallographic inversion center) into an infinite chain along the [221] direction. Benzyl ring at general position form π–π interactions with its neighbor at (1 - *x*, 1 - *y*, 1 - *z*), with distance between ring centroids of 3.534 (3) Å. This pair is further sandwiched by C—H···π(arene) hydrogen bond developed by atom C9 with H9B···*Cg* distance of 2.76 Å, and *X*—H ··· *Cg* angle of 145°, and even more weakly by the edge of the phenolic group. These aromatic interactions interspersed by the solvent molecules but propagating along the [231] direction combine with the previous linear H-bond chain to form a molecular sheet lying parallel to (-102) (Fig. 2). Orthogonally to the sheet, the third dimensionality is developed by an hydrogen bond between the amine atom N1 and the nitrate ion, along the *c* axis.

### Experimental

Diethylenetriamine (1.0311 g, 10 mmol) and salicylaldehyde (2.4408 g, 20 mmol) were dissolved in 20 ml of ethanol with few drops of glacial acetic acid. The mixture was refluxed for 3 h. On cooling, a yellow oil was isolated. To 20 ml of anhydrous methanol was added the yellow oil (1.5 g, 5.59 mmol). The mixture was cooled to 273 K before NaBH_4_ (0.63 g, 16.7 mmol) was added in small portions. A white precipitate was isolated after 30 mn of stirring by filtration. In a round-bottom flask, 15 ml of methanol and the prepared ligand (0.2 g, 0.735 mmol) were mixed. Cobalt nitrate hexahydrate (0.21, 0.735 mmol) dissolved in 5 ml of methanol was introduced. Immediate color change was observed, indicating instant occurrence of the formation of the complex. The mixture was stirred at room temperature for 2 h. The brown solution was filtered off and the filtrate was left at room temperature. After two weeks, brown crystals suitable for X-ray analyses were obtained. Yield: 75%. Anal. Calc. for [C_32_H_40_N_5_O_8_Co]: C 56.39, H 5.91, N 10.27%. Found: C 56.38, H 5.93, N 10.28%. Selected IR data (cm^-1^, KBr pellet): 400, 3216, 1600, 1582, 1458, 764.

### Refinement

Five low-resolution reflections affected by the backstop were omitted from the refinement. The refinement indicated that a water molecule and a nitrate ion reside alternatively on the same inversion site with half-occupancy. These form with the hydroxyl group a molecular H-bonded chain running in the [221] direction. Similarity restraints on 1–2 and 1–3 distances (s.u. = 0.01 and 0.04 Å, respectively) were applied to keep the nitrate ion geometry reasonable. In addition, similarity restraints on displacement parameters for the nitrate ion and water molecule (s.u. = 0.05 Å^2^), and rigid-bond restraints for anisotropic displacement parameters (s.u. = 0.01 Å^2^) in the nitrate ion were applied. Except for the water molecule, all H atoms were initially located in difference maps, then their positions were geometrically optimized and refined as riding on their parent atoms with C—H = 0.93 or 0.97 Å (aromatic CH and methylene CH_2_), N—H = 0.847–0.860 Å, and with *U*_iso_(H) = 1.2*U*_eq_(C or N), while the hydroxyl H atom was allowed to rotate about the parent C—O bond [AFIX 147 instruction in *SHELXL97* (Sheldrick, 2008)], with O2—H2O = 0.807 Å and *U*_iso_(H2O) = 1.5*U*_eq_(O2). H atoms of the water molecule were placed in calculated idealized positions using *DFIX* and DANG instructions, until being constrained with AFIX 3 instructions for the last run of refinement, in order to optimize H-bond interactions with oxygen atoms of the nitrate ion.

### Crystal data

**Table:**

[Co(C_16_H_19_N_2_O_2_)_2_]NO_3_·H_2_O	*Z* = 1
*M**_r_* = 681.62	*F*(000) = 358
Triclinic, *P*1	*D*_x_ = 1.525 Mg m^−^^3^
Hall symbol: -P 1	Mo *K*α radiation, λ = 0.71070 Å
*a* = 8.989 (3) Å	Cell parameters from 2525 reflections
*b* = 9.032 (3) Å	θ = 0.4–25.4°
*c* = 10.621 (4) Å	µ = 0.64 mm^−^^1^
α = 106.680 (2)°	*T* = 293 K
β = 99.950 (3)°	Parallelepipedic stick, brown
γ = 109.720 (2)°	0.42 × 0.14 × 0.12 mm
*V* = 742.0 (4) Å^3^	

### Data collection

**Table:**

Nonius KappaCCD diffractometer	2694 independent reflections
Radiation source: fine-focus sealed tube	2303 reflections with *I* > 2σigma(*I*)
horizontally mounted graphite crystal	*R*_int_ = 0.019
Detector resolution: 9 pixels mm^-1^	θ_max_ = 25.7°, θ_min_ = 2.8°
φ and ω scans	*h* = −10→10
Absorption correction: multi-scan (*SCALEPACK*; Otwinowski & Minor, 1997)	*k* = −10→10
*T*_min_ = 0.640, *T*_max_ = 0.930	*l* = −12→12
16124 measured reflections	

### Refinement

**Table:**

Refinement on *F*^2^	Secondary atom site location: difference Fourier map
Least-squares matrix: full	Hydrogen site location: inferred from neighbouring sites
*R*[*F*^2^ > 2σ(*F*^2^)] = 0.047	H-atom parameters constrained
*wR*(*F*^2^) = 0.126	*w* = 1/[σ^2^(*F*_o_^2^) + (0.0583*P*)^2^ + 0.3846*P*] where *P* = (*F*_o_^2^ + 2*F*_c_^2^)/3
*S* = 1.10	(Δ/σ)_max_ < 0.001
2689 reflections	Δρ_max_ = 0.25 e Å^−^^3^
234 parameters	Δρ_min_ = −0.48 e Å^−^^3^
29 restraints	Extinction correction: *SHELXL97* (Sheldrick, 2008), Fc^*^=kFc[1+0.001xFc^2^λ^3^/sin(2θ)]^-1/4^
0 constraints	Extinction coefficient: 0.058 (9)
Primary atom site location: structure-invariant direct methods	

### Fractional atomic coordinates and isotropic or equivalent isotropic displacement parameters (Å^2^)

**Table:**

	*x*	*y*	*z*	*U*_iso_*/*U*_eq_	Occ. (<1)
Co1	0.5000	0.0000	0.5000	0.0525 (2)	
O1	0.5163 (2)	0.1737 (3)	0.4297 (2)	0.0576 (5)	
O2	−0.0155 (3)	−0.4955 (3)	0.1901 (3)	0.0811 (7)	
H2O	−0.0938	−0.5812	0.1814	0.122*	
N1	0.4113 (3)	0.0896 (3)	0.6449 (2)	0.0568 (6)	
H1N	0.4568	0.0862	0.7215	0.068*	
N2	0.2647 (3)	−0.1449 (3)	0.3863 (2)	0.0568 (6)	
H2N	0.2543	−0.2386	0.3935	0.068*	
C1	0.4130 (3)	0.2510 (4)	0.4369 (3)	0.0572 (7)	
C2	0.3471 (4)	0.2816 (4)	0.3232 (3)	0.0631 (7)	
H2	0.3774	0.2503	0.2435	0.076*	
C3	0.2380 (4)	0.3575 (4)	0.3273 (4)	0.0705 (8)	
H3	0.1945	0.3762	0.2502	0.085*	
C4	0.1928 (4)	0.4059 (4)	0.4441 (4)	0.0735 (9)	
H4	0.1179	0.4561	0.4461	0.088*	
C5	0.2590 (4)	0.3796 (4)	0.5582 (4)	0.0700 (8)	
H5	0.2302	0.4149	0.6381	0.084*	
C6	0.3679 (4)	0.3016 (4)	0.5563 (3)	0.0602 (7)	
C7	0.4394 (4)	0.2700 (4)	0.6785 (3)	0.0649 (8)	
H7A	0.5575	0.3400	0.7142	0.078*	
H7B	0.3898	0.3029	0.7501	0.078*	
C8	0.2337 (3)	−0.0254 (4)	0.6089 (3)	0.0646 (8)	
H8A	0.1770	0.0346	0.6580	0.077*	
H8B	0.2245	−0.1213	0.6358	0.077*	
C9	0.1554 (3)	−0.0863 (4)	0.4564 (3)	0.0632 (8)	
H9A	0.0471	−0.1785	0.4286	0.076*	
H9B	0.1419	0.0050	0.4320	0.076*	
C10	0.2197 (3)	−0.1723 (4)	0.2372 (3)	0.0650 (8)	
H10A	0.2601	−0.2527	0.1904	0.078*	
H10B	0.2779	−0.0659	0.2277	0.078*	
C11	0.0387 (3)	−0.2355 (4)	0.1642 (3)	0.0593 (7)	
C12	−0.0188 (4)	−0.1310 (4)	0.1179 (3)	0.0673 (8)	
H12	0.0539	−0.0202	0.1376	0.081*	
C13	−0.1816 (4)	−0.1876 (5)	0.0434 (3)	0.0698 (8)	
H13	−0.2187	−0.1153	0.0138	0.084*	
C14	−0.2878 (4)	−0.3500 (4)	0.0135 (3)	0.0670 (8)	
H14	−0.3973	−0.3893	−0.0388	0.080*	
C15	−0.2359 (4)	−0.4569 (4)	0.0591 (3)	0.0644 (8)	
H15	−0.3095	−0.5679	0.0380	0.077*	
C16	−0.0727 (4)	−0.3981 (4)	0.1370 (3)	0.0602 (7)	
O11	0.7320 (11)	0.1846 (10)	0.1338 (9)	0.123 (3)	0.50
N11	0.5877 (7)	0.0882 (8)	0.0550 (6)	0.0742 (14)	0.50
O12	0.5389 (14)	0.1116 (12)	−0.0485 (10)	0.104 (3)	0.50
O13	0.5023 (13)	−0.0330 (11)	0.0776 (11)	0.097 (3)	0.50
O21	0.7119 (11)	0.2509 (10)	0.2025 (9)	0.111 (3)	0.50
H21O	0.6490	0.1443	0.2054	0.167*	0.50
H22O	0.6628	0.2429	0.1127	0.167*	0.50

### Atomic displacement parameters (Å^2^)

**Table:**

	*U*^11^	*U*^22^	*U*^33^	*U*^12^	*U*^13^	*U*^23^
Co1	0.0430 (3)	0.0580 (4)	0.0498 (3)	0.0179 (2)	0.0079 (2)	0.0177 (2)
O1	0.0520 (11)	0.0630 (12)	0.0589 (11)	0.0241 (9)	0.0139 (8)	0.0252 (9)
O2	0.0661 (14)	0.0758 (16)	0.0986 (18)	0.0311 (12)	0.0078 (13)	0.0361 (14)
N1	0.0479 (12)	0.0637 (15)	0.0515 (13)	0.0188 (11)	0.0079 (10)	0.0201 (11)
N2	0.0479 (12)	0.0655 (15)	0.0518 (13)	0.0213 (11)	0.0100 (10)	0.0201 (11)
C1	0.0450 (14)	0.0562 (16)	0.0628 (17)	0.0167 (12)	0.0093 (12)	0.0204 (13)
C2	0.0539 (16)	0.0654 (19)	0.0664 (18)	0.0210 (14)	0.0105 (13)	0.0281 (15)
C3	0.0584 (18)	0.073 (2)	0.080 (2)	0.0257 (16)	0.0103 (15)	0.0351 (17)
C4	0.0567 (18)	0.072 (2)	0.095 (3)	0.0299 (16)	0.0163 (17)	0.0352 (18)
C5	0.0600 (18)	0.0657 (19)	0.078 (2)	0.0246 (15)	0.0203 (15)	0.0201 (16)
C6	0.0527 (15)	0.0572 (17)	0.0623 (17)	0.0197 (13)	0.0107 (13)	0.0179 (13)
C7	0.0602 (17)	0.069 (2)	0.0585 (17)	0.0270 (15)	0.0133 (13)	0.0152 (14)
C8	0.0470 (15)	0.081 (2)	0.0590 (17)	0.0194 (14)	0.0154 (12)	0.0248 (15)
C9	0.0418 (14)	0.076 (2)	0.0610 (17)	0.0170 (13)	0.0119 (12)	0.0208 (15)
C10	0.0478 (15)	0.080 (2)	0.0512 (16)	0.0180 (14)	0.0062 (12)	0.0179 (14)
C11	0.0480 (15)	0.0691 (19)	0.0486 (15)	0.0186 (14)	0.0071 (11)	0.0158 (13)
C12	0.0589 (17)	0.070 (2)	0.0611 (18)	0.0168 (15)	0.0104 (14)	0.0232 (15)
C13	0.0622 (18)	0.080 (2)	0.0670 (19)	0.0297 (17)	0.0109 (15)	0.0320 (17)
C14	0.0483 (15)	0.087 (2)	0.0565 (17)	0.0240 (16)	0.0074 (13)	0.0230 (16)
C15	0.0514 (16)	0.0666 (19)	0.0599 (17)	0.0169 (14)	0.0086 (13)	0.0155 (14)
C16	0.0531 (16)	0.0650 (18)	0.0565 (16)	0.0243 (14)	0.0101 (12)	0.0178 (14)
O11	0.129 (6)	0.083 (5)	0.122 (7)	0.026 (4)	−0.011 (5)	0.041 (5)
N11	0.070 (3)	0.073 (4)	0.079 (4)	0.027 (3)	0.021 (3)	0.032 (3)
O12	0.113 (7)	0.107 (7)	0.078 (5)	0.028 (6)	0.011 (4)	0.044 (5)
O13	0.094 (5)	0.089 (6)	0.090 (5)	0.012 (4)	0.018 (4)	0.042 (5)
O21	0.100 (5)	0.081 (5)	0.134 (7)	0.017 (4)	0.016 (5)	0.050 (5)

### Geometric parameters (Å, °)

**Table:**

Co1—O1^i^	1.896 (2)	N11—O13	1.222 (9)
Co1—O1	1.896 (2)	N11—O13^ii^	1.348 (12)
Co1—N1^i^	1.950 (2)	N11—N11^ii^	1.728 (12)
Co1—N1	1.950 (2)	N11—O12^ii^	1.753 (10)
Co1—N2^i^	1.997 (2)	O12—O13^ii^	0.627 (11)
Co1—N2	1.997 (2)	O12—N11^ii^	1.753 (10)
O1—C1	1.337 (3)	O13—O12^ii^	0.627 (11)
O2—C16	1.358 (4)	O13—N11^ii^	1.348 (12)
N1—C7	1.484 (4)	O2—H2O	0.8200
N1—C8	1.488 (4)	N1—H1N	0.8595
N2—C9	1.475 (4)	N2—H2N	0.8468
N2—C10	1.490 (4)	C2—H2	0.9300
C1—C2	1.393 (4)	C3—H3	0.9300
C1—C6	1.397 (4)	C4—H4	0.9300
C2—C3	1.373 (4)	C5—H5	0.9300
C3—C4	1.370 (5)	C7—H7A	0.9700
C4—C5	1.375 (5)	C7—H7B	0.9700
C5—C6	1.385 (4)	C8—H8A	0.9700
C6—C7	1.493 (4)	C8—H8B	0.9700
C8—C9	1.502 (4)	C9—H9A	0.9700
C10—C11	1.502 (4)	C9—H9B	0.9700
C11—C12	1.381 (4)	C10—H10A	0.9700
C11—C16	1.381 (4)	C10—H10B	0.9700
C12—C13	1.375 (4)	C12—H12	0.9300
C13—C14	1.359 (5)	C13—H13	0.9300
C14—C15	1.371 (5)	C14—H14	0.9300
C15—C16	1.386 (4)	C15—H15	0.9300
O11—N11	1.259 (9)	O21—H21O	0.9503
N11—O12	1.214 (11)	O21—H22O	0.9504
			
O1^i^—Co1—O1	180	O12—N11—O11	118.7 (8)
O1^i^—Co1—N1^i^	94.28 (10)	O13—N11—O11	120.6 (8)
O1—Co1—N1^i^	85.72 (10)	C16—O2—H2O	109.5
O1^i^—Co1—N1	85.72 (9)	C7—N1—H1N	104.3
O1—Co1—N1	94.28 (10)	C8—N1—H1N	104.2
N1^i^—Co1—N1	180	Co1—N1—H1N	111.3
O1^i^—Co1—N2^i^	93.87 (9)	C9—N2—H2N	107.1
O1—Co1—N2^i^	86.13 (9)	C10—N2—H2N	108.3
N1^i^—Co1—N2^i^	86.32 (10)	Co1—N2—H2N	100.6
N1—Co1—N2^i^	93.68 (9)	C3—C2—H2	119.6
O1^i^—Co1—N2	86.13 (9)	C1—C2—H2	119.6
O1—Co1—N2	93.87 (9)	C4—C3—H3	119.8
N1^i^—Co1—N2	93.68 (9)	C2—C3—H3	119.8
N1—Co1—N2	86.32 (10)	C3—C4—H4	120.3
N2^i^—Co1—N2	180	C5—C4—H4	120.3
C1—O1—Co1	122.22 (18)	C4—C5—H5	119.4
C7—N1—C8	113.1 (2)	C6—C5—H5	119.4
C7—N1—Co1	114.85 (19)	N1—C7—H7A	109.1
C8—N1—Co1	108.49 (18)	C6—C7—H7A	109.1
C9—N2—C10	112.6 (2)	N1—C7—H7B	109.1
C9—N2—Co1	109.04 (17)	C6—C7—H7B	109.1
C10—N2—Co1	117.92 (18)	H7A—C7—H7B	107.9
O1—C1—C2	119.8 (3)	N1—C8—H8A	109.8
O1—C1—C6	121.5 (3)	C9—C8—H8A	109.8
C2—C1—C6	118.6 (3)	N1—C8—H8B	109.8
C3—C2—C1	120.8 (3)	C9—C8—H8B	109.8
C4—C3—C2	120.5 (3)	H8A—C8—H8B	108.3
C3—C4—C5	119.5 (3)	N2—C9—H9A	110.1
C4—C5—C6	121.2 (3)	C8—C9—H9A	110.1
C5—C6—C1	119.4 (3)	N2—C9—H9B	110.1
C5—C6—C7	122.0 (3)	C8—C9—H9B	110.1
C1—C6—C7	118.7 (3)	H9A—C9—H9B	108.4
N1—C7—C6	112.3 (2)	N2—C10—H10A	108.2
N1—C8—C9	109.2 (2)	C11—C10—H10A	108.2
N2—C9—C8	108.0 (2)	N2—C10—H10B	108.2
N2—C10—C11	116.5 (2)	C11—C10—H10B	108.2
C12—C11—C16	118.2 (3)	H10A—C10—H10B	107.3
C12—C11—C10	119.6 (3)	C13—C12—H12	119.3
C16—C11—C10	122.1 (3)	C11—C12—H12	119.3
C13—C12—C11	121.3 (3)	C14—C13—H13	120.3
C14—C13—C12	119.4 (3)	C12—C13—H13	120.3
C13—C14—C15	121.0 (3)	C13—C14—H14	119.5
C14—C15—C16	119.4 (3)	C15—C14—H14	119.5
O2—C16—C11	117.3 (3)	C14—C15—H15	120.3
O2—C16—C15	122.2 (3)	C16—C15—H15	120.3
C11—C16—C15	120.6 (3)	H21O—O21—H22O	104.1
O12—N11—O13	120.6 (8)		
			
N1^i^—Co1—O1—C1	149.8 (2)	C7—N1—C8—C9	90.2 (3)
N1—Co1—O1—C1	−30.2 (2)	Co1—N1—C8—C9	−38.5 (3)
N2^i^—Co1—O1—C1	−123.6 (2)	C10—N2—C9—C8	−167.3 (3)
N2—Co1—O1—C1	56.4 (2)	Co1—N2—C9—C8	−34.4 (3)
O1^i^—Co1—N1—C7	161.25 (19)	N1—C8—C9—N2	48.1 (3)
O1—Co1—N1—C7	−18.75 (19)	C9—N2—C10—C11	−33.9 (4)
N2^i^—Co1—N1—C7	67.64 (19)	Co1—N2—C10—C11	−162.3 (2)
N2—Co1—N1—C7	−112.36 (19)	N2—C10—C11—C12	112.2 (3)
O1^i^—Co1—N1—C8	−71.16 (19)	N2—C10—C11—C16	−70.1 (4)
O1—Co1—N1—C8	108.84 (19)	C16—C11—C12—C13	−1.8 (5)
N2^i^—Co1—N1—C8	−164.77 (19)	C10—C11—C12—C13	176.0 (3)
N2—Co1—N1—C8	15.23 (19)	C11—C12—C13—C14	−0.7 (5)
O1^i^—Co1—N2—C9	96.9 (2)	C12—C13—C14—C15	1.5 (5)
O1—Co1—N2—C9	−83.1 (2)	C13—C14—C15—C16	0.0 (5)
N1^i^—Co1—N2—C9	−169.1 (2)	C12—C11—C16—O2	−176.4 (3)
N1—Co1—N2—C9	10.9 (2)	C10—C11—C16—O2	5.8 (4)
O1^i^—Co1—N2—C10	−133.0 (2)	C12—C11—C16—C15	3.4 (4)
O1—Co1—N2—C10	47.0 (2)	C10—C11—C16—C15	−174.4 (3)
N1^i^—Co1—N2—C10	−39.0 (2)	C14—C15—C16—O2	177.2 (3)
N1—Co1—N2—C10	141.0 (2)	C14—C15—C16—C11	−2.5 (5)
Co1—O1—C1—C2	−136.5 (2)	O13—N11—O12—O13^ii^	−29 (3)
Co1—O1—C1—C6	43.1 (3)	O11—N11—O12—O13^ii^	147 (2)
O1—C1—C2—C3	178.6 (3)	N11^ii^—N11—O12—O13^ii^	−18 (2)
C6—C1—C2—C3	−1.0 (5)	O12^ii^—N11—O12—O13^ii^	−18 (2)
C1—C2—C3—C4	0.5 (5)	O13—N11—O12—N11^ii^	−10.6 (13)
C2—C3—C4—C5	0.7 (5)	O11—N11—O12—N11^ii^	164.8 (9)
C3—C4—C5—C6	−1.5 (5)	O13^ii^—N11—O12—N11^ii^	18 (2)
C4—C5—C6—C1	0.9 (5)	O12^ii^—N11—O12—N11^ii^	0.0
C4—C5—C6—C7	−179.2 (3)	O12—N11—O13—O12^ii^	49 (5)
O1—C1—C6—C5	−179.3 (3)	O11—N11—O13—O12^ii^	−127 (4)
C2—C1—C6—C5	0.3 (4)	O13^ii^—N11—O13—O12^ii^	36 (4)
O1—C1—C6—C7	0.8 (4)	N11^ii^—N11—O13—O12^ii^	36 (4)
C2—C1—C6—C7	−179.6 (3)	O12—N11—O13—N11^ii^	12.9 (16)
C8—N1—C7—C6	−67.3 (3)	O11—N11—O13—N11^ii^	−162.4 (9)
Co1—N1—C7—C6	57.9 (3)	O13^ii^—N11—O13—N11^ii^	0.0
C5—C6—C7—N1	126.1 (3)	O12^ii^—N11—O13—N11^ii^	−36 (4)
C1—C6—C7—N1	−54.0 (4)		

Symmetry codes: (i) −*x*+1, −*y*, −*z*+1; (ii) −*x*+1, −*y*, −*z*.

### Hydrogen-bond geometry (Å, °)

**Table:**

*D*—H···*A*	*D*—H	H···*A*	*D*···*A*	*D*—H···*A*
O21—H21O···O12^ii^	0.95	2.27	3.025 (12)	136.
O21—H22O···O13^ii^	0.95	2.18	2.922 (13)	134.
O2—H2O···O21^iii^	0.82	1.99	2.787 (9)	165.
O2—H2O···O11^iii^	0.82	2.01	2.823 (9)	169.
N1—H1N···O12^iv^	0.86	2.35	3.185 (10)	165.
N1—H1N···O13^i^	0.86	2.31	3.145 (11)	163.

Symmetry codes: (ii) −*x*+1, −*y*, −*z*; (iii) *x*−1, *y*−1, *z*; (iv) *x*, *y*, *z*+1; (i) −*x*+1, −*y*, −*z*+1.
